# Inflammatory phenotyping predicts clinical outcome in COVID-19

**DOI:** 10.1186/s12931-020-01511-z

**Published:** 2020-09-22

**Authors:** H. Burke, A. Freeman, D. C. Cellura, B. L. Stuart, N. J. Brendish, S. Poole, F. Borca, H. T. T. Phan, N. Sheard, S. Williams, C. M. Spalluto, K. J. Staples, T. W. Clark, T. M. A. Wilkinson, on behalf of the REACT COVID investigators, Tom Wilkinson, Anna Freeman, Hannah Burke, Ahilanadan Dushianthan, Michael Celinski, James Batchelor, Saul N. Faust, Gareth Thomas, Christopher Kipps

**Affiliations:** 1 School of Clinical and Experimental Sciences, Faculty of Medicine, University of Southampton, Southampton General Hospital, LF13A, South Academic Block, Southampton, SO16 6YD UK; 2 University Hospitals Southampton NHS Foundation Trust, Southampton, UK; 3 Southampton Clinical Trials Unit, University of Southampton, Southampton, UK; 4 NIHR Southampton Biomedical Research Centre, University Hospital Southampton NHS Foundation Trust, Southampton, UK; 5 Clinical Informatics Research Unit Faculty of Medicine, University of Southampton, Southampton, UK; 6 Wessex Investigational Sciences Hub, University Of Southampton, University Hospital Southampton NHS Foundation Trust, Southampton, UK; 7 NIHR Post-Doctoral Fellowship Programme, Southampton, UK

**Keywords:** COVID-19, SARS-CoV-2, IL-33, TNF-α, Point-of-care testing

## Abstract

**Background:**

The COVID-19 pandemic has led to more than 760,000 deaths worldwide (correct as of 16th August 2020). Studies suggest a hyperinflammatory response is a major cause of disease severity and death. Identitfying COVID-19 patients with hyperinflammation may identify subgroups who could benefit from targeted immunomodulatory treatments. Analysis of cytokine levels at the point of diagnosis of SARS-CoV-2 infection can identify patients at risk of deterioration.

**Methods:**

We used a multiplex cytokine assay to measure serum IL-6, IL-8, TNF, IL-1β, GM-CSF, IL-10, IL-33 and IFN-γ in 100 hospitalised patients with confirmed COVID-19 at admission to University Hospital Southampton (UK). Demographic, clinical and outcome data were collected for analysis.

**Results:**

Age > 70 years was the strongest predictor of death (OR 28, 95% CI 5.94, 139.45). IL-6, IL-8, TNF, IL-1β and IL-33 were significantly associated with adverse outcome. Clinical parameters were predictive of poor outcome (AUROC 0.71), addition of a combined cytokine panel significantly improved the predictability (AUROC 0.85). In those ≤70 years, IL-33 and TNF were predictive of poor outcome (AUROC 0.83 and 0.84), addition of a combined cytokine panel demonstrated greater predictability of poor outcome than clinical parameters alone (AUROC 0.92 vs 0.77).

**Conclusions:**

A combined cytokine panel improves the accuracy of the predictive value for adverse outcome beyond standard clinical data alone. Identification of specific cytokines may help to stratify patients towards trials of specific immunomodulatory treatments to improve outcomes in COVID-19.

## Background

Coronavirus disease 2019 (COVID-19) has been confirmed in 21,294,845 people worldwide as of 16th August 2020, carrying a mortality of nearly 4%, compared with a mortality rate of less than 1% from influenza [1]. Although this mortality rate maybe an overestimation due to lack of adequate testing among other factors, currently more than 760,000 deaths are attributed to COVID-19 and therefore there is an urgent need for effective treatment.

Accumulating evidence suggests that in addition to direct viral damage, uncontrolled inflammation contributes to disease severity in COVID-19 [2]. This is characterised by a profound cytokine response in the host with raised levels of inflammatory mediators leading to tissue damage and multi-organ failure [3–5]. So far, the only treatment to demonstrate mortality benefit in COVID-19 randomised clinical trials is dexamethasone, but the mechanism through which this effect is mediated is unclear, with dexamethasone dampening inflammation in a non-specific manner [6]. In addition, corticosteroids may also increase the risk of secondary infection in COVID-19 [7]. Therefore, several studies trialling more targeted immunomodulatory approaches in COVID-19 to suppress inflammatory responses are in progress [8].

Disease patterns and responses to acute SARS-CoV-2 infection appear heterogenous and importantly patients with severe COVID-19 present with differential cytokine patterns [3]. This may be driven by varying immunological responses to the SARS-CoV-2, and endotyping of these responses is a key step towards precision medicine in this disease. Finally, targeting immunomodulatory therapeutic agents will limit toxicity and side effects associated with these agents.

We hypothesise that assessment of inflammatory cytokines in patients with COVID-19 at presentation to hospital may identify those likely to deteriorate and develop severe disease. Furthermore, understanding the variation in cytokine profiles at presentation may give insights into which subgroups may benefit from immunomodulatory therapy.

We aim to demonstrate that an accurate point-of-care test (POCT) to diagnose SARS-CoV-2 infection quickly at presentation, in combination with a cytokine assay can identify patients at risk of deterioration. This could facilitate transition towards a precision medicine approach to COVID-19 and highlight those subgroups who may benefit from targeted immunomodulatory treatments earlier in their disease process.

## Methods

### Study design and participants

This study was nested within the CoV-19POC study, a trial assessing the clinical impact of molecular POCT in patients within 24 h of presentation to University Hospital Southampton (UHSFT), a tertiary centre located in Southampton, United Kingdom (UK), with suspected COVID-19. Any adult aged 18 years old and over presenting to hospital with suspected COVID-19 and/or an acute respiratory illness was eligible for the study. Full details of the inclusion and exclusion criteria can be found in the master protocol, linked below. The study was approved by the South-Central Hampshire A Research Ethics Committee: REC reference 20/SC/0138, on the 16th March 2020 [9].

### Procedures

Combined nose and throat swabs were obtained from patients at admission to UHSFT and were inactivated by placing them directly in media containing guanidine thiocyanate and detergent. They were then tested for SARS-CoV-2 using the CE marked QIAstat-Dx® Respiratory SARS-CoV Panel (Qiagen™, Manchester, UK), in a dedicated POCT hub embedded within the medical admissions unit. Results were available after 70 min.

Blood was obtained by venesection in serum separating tubes. Samples were left to clot for at least 30 min prior to centrifugation (2000G for 10 min) in line with manufacturers instructions. Serum aliquots were frozen on the day of venesection at − 80 °C in a fully licensed biorepository, and thawed once for later testing. Sera were aliquoted and handled under containment level 2 conditions within a suitable microbiological safety cabinet in line with Public Health England guidance. Serum samples were not inactivated prior to testing.

Demographic and clinical data were collected at enrolment and intervention and outcome data were collected retrospectively from case notes and electronic healthcare records. To capture how unwell patients were at enrolment into the study, the National Early Warning Score 2 (NEWS2), comprising respiratory rate, oxygen saturations, temperature, systolic blood pressure, pulse rate and level of consciousness, was recorded for each patient as a summary of physiological measurements [10]. Laboratory (other than the cytokine readouts) and treatment data, was collected using a real-time data analytics tool (REal-time Analytics for Clinical Trials; digital Experimental Medicines Team, Manchester, UK).

All patients recruited into the main CoV-19POC study who tested positive for SARS-CoV-2 (*n* = 193) were eligible for serum sampling, but a sample was only collected in 100 patients. The patients who were not tested (*n* = 93) were either already discharged from hospital by the time of their result, declined testing, or the research team were unable to obtain a sample from venesection. Therefore, in this sub-study, 100 SARS-CoV-2 positive patients had serum processed for cytokine analysis. These results were not known to the treating clinician and therefore did not bias treatment strategy.

### Cytokine analysis

Serum from 100 COVID-19 patients were analysed for the following inflammatory cytokines: interleukin (IL)-6, IL-8, IL-1β, IL-33, IL-10, Tumour necrosis factor alpha (TNF), granulocyte-macrophage colony-stimulating factor (GM-CSF) and interferon (IFN) –γ using the Ella™ platform (ProteinSimple, San Jose, California, US) following the manufacturer’s instructions. This cytokine detection system is based on four multiplexed microfluidics enzyme-linked immunosorbent assays (ELISA) run in triplicate within a cartridge. The cytokine panel was chosen based on review of emerging literature on inflammatory responses in COVID-19 [2, 11].

For this assay, 25 μL of serum was combined with 25 μL of assay-specific diluent, to achieve a 1:2 dilution of the original sample. A quality control was run in each cartridge alongside the samples to be tested. Results were scrutinised and any samples with coefficient of variation higher than 10% between the triplicates were re-run. Analytical validation for the lower cytokine ranges was performed using reference cytokine controls for a 7-point standard curve with known quantities, and the Ella™ measurements were comparable above 0.1 pg/mL (data not shown).

### Study outcomes

The composite end point was admission to the intensive care, the use mechanical ventilation, and/or death. These outcomes were used in a previous observational studies to assess the severity of infectious diseases, such as H7N9 infection [12] and SARS CoV-2 infection [13].

### Sample size

A pragmatic sample size for this sub-study was driven by the access to patient samples and the availability of the Ella™ cytokine analysis platform.

### Statistical analysis

Baseline patient characteristics were summarised using standard descriptive statistics, with number and percentages for binary and categorical outcomes and appropriate measures for continuous outcomes – means and standard deviations for normally distributed variables and medians and lower and upper quartiles for skewed distributions. Cytokine readouts were standardised by calculating a z-score to allow comparison between samples. Spearman’s correlation coefficients were used to quantify the correlations between cytokines and continuous variables. Mann Whitney U tests were used to test whether there were significant differences by sociodemographic factors in cytokine levels. Logistic regression models were used to explore the relationship between the outcome and independent variables. Models are presented for univariate analyses and for multivariable analyses for each cytokine. The covariates in the multivariable models were prespecified and include gender, age, NEWS2 Score, comorbid conditions, treatment medications (e.g. systemic glucocorticoids) and smoking status. Model discrimination is presented using the area under the receiver operator characteristic curve (AUROC). Given the small sample size and the possibility of overfitting in the model, we have used bootstrapped estimates in 1000 bootstrapped samples to estimate the AUROC.

## Results

### Cohort characteristics

Clinical and laboratory data were collected as part of CoV-19POC study from 100 patients with PCR confirmed SARS-CoV-2 infection who were admitted to UHSFT (Southampton, UK) between 20th March and 29th April 2020. The median time from positive POCT for SARS-CoV-2 to blood sampling was 3 h 12 min (IQR 59 mins to 17 h 18 mins).

Patient clinical characteristics are listed in Table 1. Patients recruited to the study were predominantly male (61%), current non-smokers (82%) with a mean age of 61.55 years. Most were of white, British ethnicity (73.2%) and the most common comorbidities recorded were hypertension (40.6%), chronic respiratory disease (27.7%), diabetes (20.8%) and cardiac disease (19.8%). The median duration of symptoms at admission to hospital was 7 days. Patients recruited to the study were acutely unwell at presentation with a mean NEWS2 score of 5.2 corresponding to a medium risk of deterioration and prompting an urgent doctor review. Markers of inflammation (C-reactive protein, CRP; lactate dehydrogenase, LDH; Ferritin and D-dimer) were raised. In terms of treatment received, 63 (62.4%) received intravenous antibiotics and 10 (9.9%) received systemic glucocorticoids. Of the total cohort (*n* = 100), 18 (20.7%) patients died, and 44 (43.6%) patients achieved the composite end point.

**Table 1:** Clinical Characteristics

	Total Cohort (*n* = 100)	Presence Composite End Point (*n* = 44)	Univariate Odds Ratio (95% CI) Composite End Point	Death ^†^ (*n* = 18)	Univariate Odds Ratio (95% CI) Death
Patient demographics					
Sex					
- Male	61 (61%)	29 (65.9%)	1.41 (0.62, 3.18)	11 (61.1%)	1.01 (0.35, 2.93)
- Female	39 (39%)	15 (34.1%)	^a^REF	7 (38.9%)	^a^REF
Mean age (±SD)	61.55 (17.28)	63.07 (17.91)	1.01 (0.99, 1.03)	80.89 (9.98)	1.15 (1.07, 1.23)
Age ≥ 70 years old	35 (35%)	16 (36.6%)	1.14 (0.50, 2.61)	16 (88.9%)	**28 (5.94, 139.45)**
Age < 70 years old	65 (65%)	28 (63.7%)	^a^REF	2 (11.1%)	^a^**REF**
Current smoker					
- Yes	5 (5%)	2 (4.6%)	0.81 (0.13, 5.11)	1 (5.6%)	1.21 (0.12, 11.79)
- No	82 (82%)	37 (84.1%)	^a^REF	12 (66.7%)	^a^REF
- Unknown	13 (13%)	5 (11.4%)	0.68 (0.21, 2.19)	5 (27.8%)	3.45 (0.94, 12.73)
Ethnicity*					
- White	71 (73.2%)	30 (69.8%)	0.73 (0.30, 1.80)	15 (88.2%)	3.5 (0.73, 16.72)
- Black and ethnic minorities	26 (26.8%)	13 (30.2%)	^a^REF	2 (11.8%)	^a^REF
Past Medical history					
Hypertension	41 (40.6%)	19 (43.2%)	1.21 (0.54, 2.69)	13 (72.2%)	**5.56 (1.76, 17.52)**
Cardiac disease	20 (19.8%)	9 (20.5%)	1.07 (0.40, 2.88)	8 (44.4%)	**5.33 (1.67, 17.09)**
COPD	10 (9.9%)	7 (15.9%)	3.41 (0.83, 14.02)	5 (27.8%)	**8.46 (1.80, 39.87)**
Asthma	17 (16.8%)	8 (18.2%)	1.19 (0.42, 3.37)	4 (22.2%)	1.51 (0.42, 5.44)
Chronic Respiratory Disease	28 (27.7%)	15 (34.1%)	1.75 (0.73, 4.21)	10 (55.6%)	**4.5 (1.51, 13.41)**
Chronic Kidney disease	7 (6.9%)	4 (9.1%)	1.80 (0.38, 8.50)	3 (16.7%)	4.40 (0.81, 23.98)
Liver Disease	3 (3.0%)	2 (4.6%)	2.67 (0.23, 30.40)	0 (0.00%)	NA
Diabetes	21 (20.8%)	13 (29.6%)	2.46 (0.91, 6.63)	9 (50.0%)	**5.70 (1.82, 17.87)**
Malignancy	8 (7.9%)	4 (9.1%)	1.33 (0.31, 5.62)	4 (22.2%)	**4.64 (1.03, 20.84)**
Dementia	11 (11.0%)	5 (11.4%)	1.07 (0.30, 3.76)	5 (27.8%)	**4.85 (1.22, 19.18)**
Presentation to Hospital					
Median day of presentation (LQ, UQ)	7 (3,10)	7 (3, 9.5)	0.95 (0.89, 1.03)	3 (1,7)	0.80 (0.69, 0.93)
NEWS2 Score at admission to hospital Mean (±SD)	5.2 (2.76)	6.15 (2.32)	**1.27 (1.07, 1.50)**	5.19 (2.50)	1.05 (0.85, 1.30)
Admission COVID-19 Blood panel					
Neutrophil count 10^9^L Median (IQR)	6.44 (3.54)	7.83 (3.57)	**1.25 (1.09, 1.43)**	8.31 (4.00)	**1.22 (1.05, 1.42)**
Lymphocyte count 10^9^L Median (IQR)	1.56 (3.82)	1.03 (0.50)	0.87 (0.61, 1.23)	0.92 (0.50)	0.41 (0.10, 1.63)
Neutrophil/Lymphocyte ratio Median (IQR)	12 (5, 12)	22 (9, 67)	1.14 (1.05, 1.24)	48 (21, 1028)	**1.20 (1.08, 1.33)**
C-Reactive Protein mg/L Median (IQR)	108 (26, 153)	131 (62, 165)	1.01 (1.00, 1.02)	52 (13, 209)	1.01 (0.99, 1.01)
Ferritin μg/L Median (IQR)	625 (286, 1366)	1075 (523, 2002)	1.00 (1.00, 1.00)	602 (246, 2017)	1.00 (0.99, 1.00)
Lactate dehydrogenase U/L Median (IQR)	752 (548, 998)	874 (704, 1081)	1.00 (1.00, 1.00)	833 (759. 946)	1.00 (0.99, 1.00)
D-dimer ng/mL Median (IQR)	444 (276, 720)	527 (298, 1110)	1.00 (0.99, 1.00)	563 (374, 1682)	1.00 (0.99, 1.01)
High sensitivity cardiac Troponin I ng/L Median (IQR)	5.4 (3.6, 5.4)	7.7 (4.8, 15.4)	1.00 (0.99, 1.01)	10.3 (6, 15.8)	1.00 (1.00, 1.01)
Treatments and Outcomes					
Intravenous antibiotics	63 (62.4%)	27 (61.4%)	0.88 (0.39, 2.00)	14 (77.8%)	**1.87 (0.55, 6.30)**
Systemic glucocorticoids	10 (9.9%)	6 (13.6%)	2.05 (0.54, 7.78)	5 (27.8%)	**6.25 (1.48, 26.47)**
Intensive Care Admission	28 (27.7%)				
Mechanical ventilation					
- Invasive	18 (17.8%)				
- Non-invasive^*^	21 (21.7%)				
Death^†^	18 (20.7%)				
Composite End Point^b^	44 (43.6%)				

^*^N = 97; ^**†**^*N* = 87. Cohort numbers vary, due to missing data or as some patients are yet to be discharged from ICU and/or hospital

^a^REF indicates the reference category which is the category of comparison in that section

^b^Composite end point was admission to the intensive care, the use mechanical ventilation, and/or death

Older age was significantly associated with death. This was a large effect size with those aged 70 years or over having a univariate odds ratio (OR) of 28 (95% confidence intervals (CI) 5.94, 139.45) of death. In addition, several comorbidities including hypertension, cardiac disease, COPD, diabetes, dementia and malignancy were also associated with death. The only variable significant on univariate analysis for the composite outcome was higher NEWS2 score (OR 1.27, 95% CI 1.07, 1.50). When looking at standard clinical blood tests, neutrophil count at admission, and neutrophil:lymphocyte ratio were both independently predictive of death, with a univariate OR of 1.22 (95% CI 1.05, 1.42) and 1.20 (95% CI 1.08, 1.33) respectively. The results for treatment with steroids as a predictor for poor outcome (univariate OR 6.25, 95% CI 1.48, 26.47) is likely due to steroids being given mainly in the setting of intensive care. Patients who were given steroids were more unwell prior to their treatment rather than treatment resulting in worse outcome. Treatment strategies also had no bearing on cytokine levels as serum was taken at admission prior to escalation of treatment.

### Association between serum cytokine levels on clinical outcomes in COVID-19

Patient serum cytokine levels measured on admission to hospital are listed in Table 2. On both univariate and adjusted models TNF, IL-6, IL-8, IL-1β and IL-33 were significantly associated with the composite end point, with the biggest effect size seen in IL-1β and IL-33 (OR 10.21, 95% CI 1.11, 93.37 and OR 4.78, 95% CI 1.18, 19.30 respectively). Standard demographic information and NEWS2 score was reasonably accurate at predicting worse outcome, with an AUROC of 0.71. This improved to 0.81 with the addition of the COVID-19 blood panel (comprised of neutrophils, lymphocytes, neutrophil:lymphocyte ratio, CRP, LDH, ferritin, D Dimer and high sensitivity (HS) cardiac troponin I. However, addition of the cytokine panel to demographic and NEWS2 score improved the model to a greater degree than COVID-19 blood panel with an AUROC of 0.85. A combination of the demographic and NEWS2 data, COVID-19 panel blood and cytokine levels further improved the predictive capability of the model, giving an AUROC of 0.91 (see Table 2).

**Table 2:** Serum cytokine levels for patients at admission to hospital

Serum cytokines^a^ (pg/mL)	Total cohort, *N* = 100 Median (LQ,UQ)	Presence composite end point^b^ Median (LQ, UQ)	OR (95% CI) for composite end point^b^	Adjusted^c^ OR (95% CI) for composite end point^b^	AUROC for Adjusted model^c^
Model with demographics +NEWS2 only					0.71 (0.60, 0.81)
Model with COVID-19 blood panel^d^					0.81 (0.86, 0.91)
IL-6	48.2 (28.5, 96.5)	88.5 (43.0, 202)	**1.01 (1.00, 1.02)**	**1.01 (1.00, 1.02)**	**0.81 (0.72, 0.89)**
TNF-α	19.7 (15.2, 24.8)	21.5 (17.4, 27.1)	**1.03 (1.00, 1.07)**	**1.02 (0.98, 1.07)**	0.77 (0.66, 0.85)
IL-8	36.4 (25.6, 58.0)	49.9 (32.7, 85.8)	**1.03 (1.01, 1.04)**	**1.02 (1.00, 1.04)**	0.78 (0.68, 0.86)
IL-1β	0.4 (0.2, 0.6)	0.5 (0.3, 0.7)	**10.86 (1.82, 64.84)**	**10.21 (1.11, 93.37)**	0.78 (0.68, 0.87)
GM-CSF	1.4 (0.8, 2.2)	1.4 (0.8, 2.2)	1.03 (0.97, 1.11)	1.02 (0.96, 1.10)	0.73 (0.62, 0.82)
IFN-γ	12.2 (3.7, 27.4)	11.1 (3.7, 35.2)	1.00 (0.99, 1.01)	1.00 (0.99, 1.01)	0.74 (0.64, 0.84)
IL-33	0.4 (0.2, 0.6)	0.5 (0.3, 0.8)	**6.48 (1.79, 23.47)**	**4.78 (1.18, 19.30)**	**0.80 (0.68, 0.87)**
IL-10	15.2 (9.1, 26.7)	19.2 (14.5, 31.8)	1.02 (0.99, 1.03)	1.01 (0.99, 1.03)	0.74 (0.64, 0.83)
Model with all cytokines					**0.85 (0.77, 0.92)**
Model with all cytokines and COVID-19 blood panel^d^					**0.91 (0.81, 0.98)**

LQ = lower quartile, is the 25% point of the data

UQ = upper quartile is the 75% point of the data

Significant results (AUROC> 0.8) in bold

^a^All cytokine values have been standardised before including in the model ^b^Composite end point was admission to the intensive care, the use mechanical ventilation, and/or death

^c^Adjusted models control for gender, age, NEWS2 Score, treatment, comorbid conditions and smoking status

^d^COVID-19 blood panel comprised neutrophils, lymphocytes, neutrophil:lymphocyte ratio, C-reactive protein, Lactate Dehydrogenase, D dimer, Ferritin and high sensitivity cardiac troponin I

When comparing the cytokine levels with patient characteristics, there were no differences in cytokine levels between male and female participants (see Table E1 in the online data supplement). Furthermore, cytokine levels did not seem to differ with age except for TNF, which was significantly elevated in patients aged 70 years or over (*p* = 0.006) (see Table E2 in the online data supplement). Cytokines IL-6 and TNF showed moderate correlation with CRP (R^2^ 0.45 and 0.43 respectively), with IL-6 also showing a moderate correlation with LDH. Serum IL-8 levels correlated moderately with LDH and D-dimer levels (R^2^ 0.52 and 0.47 respectively) (see Table E3 in the online data supplement).

### Comparison between cytokines and standard clinical measurements on outcomes in COVID-19

We assessed whether cytokine levels were predictive of adverse outcome to a greater degree than standard clinical measurements. Looking at the whole cohort, increased age was the greatest predictor for death (See Table 1, OR 28, 95% CI 5.94, 139.4), and in combination with other baseline demographics gave an AUROC for death of 0.93 (see Table E4 in the online data supplement). The addition of individual cytokine data was no more predictive than the use of standard clinical tests (see Table E4 in the online data supplement). Therefore, given increased age is a fixed variable that could not be modified by potential treatment, we evaluated cytokine levels as predictors of outcome in those under 70 years, given this is the cut off for shielding in the UK. Cytokines TNF and IL-33 were independent predictors of the composite end point with an AUROC for adjusted model of 0.84 (*p* = 0.02) and 0.83 (*p* = 0.03) respectively, adding a greater degree of prediction than that of the demographic model and NEWS2 score alone (AUROC 0.77, see Table 3). Whilst clinical blood tests were also predictive of poor outcome in combination (AUROC of 0.88), they did not improve prediction of poor outcome individually in the same way as TNF and IL-33, with neutrophil count as the only significant predictor of poor outcome (OR 1.25, 95% CI 1.09, 1.43, see Table 1.). Furthermore, when using all the cytokines combined with NEWS2 and demographic data, the AUROC for the adjusted model improved further to 0.92. The under 70 years subcohort was too small to provide interpretable results when demographic data, NEWS2 and standard clinical COVID-19 blood panel were used in combination with the cytokine results.

**Table 3:** Associations between cytokine levels and poor outcome in COVID-19 in patients under 70 years old (*N* = 66)

	OR (95% CI) for composite end point^c^	Adjusted^b^ OR (95% CI) for composite end point^c^	AUROC for Adjusted model^b^
Model with demographics +NEWS2 only			0.77 (0.64, 0.87)
Model with COVID-19 blood panel^d^			0.88 (0.74, 0.97)
IL-6	1.01 (0.99, 1.02)	1.01 (0.99, 1.02)	0.79 (0.65, 0.88)
TNF	**1.12 (1.03, 1.22)**	**1.17 (1.03, 1.35)**	**0.84 (0.73, 0.92)**
IL-8	1.02 (1.00, 1.05)	1.01 (0.98, 1.03)	0.78 (0.66, 0.89)
IL-1β	9.20 (0.97, 87.40)	19.75 (0.78, 497.78)	0.82 (0.70, 0.92)
GM-CSF	0.96 (0.84, 1.10)	0.93 (0.78, 1.11)	0.77 (0.63, 0.87)
IFN-γ	0.99 (0.98, 1.01)	1.00 (0.98, 1.03)	0.77 (0.64, 0.88)
IL-33	**13.03 (2.03, 83.72)**	**11.14 (1.01, 123.72)**	**0.83 (0.72, 0.91)**
IL-10	1.01 (0.99, 1.02)	1.00 (0.98, 1.02)	0.77 (0.64, 0.87)
Model with all cytokines^a^ included			**0.92 (0.83, 0.97)**

Significant results (AUROC> 0.8) in bold

^a^All cytokine values have been standardised before including in the model

^b^Adjusted models control for gender, age, NEWS2 Score, comorbid conditions and smoking status

^c^Composite end point was admission to the intensive care, the use mechanical ventilation, and/or death

^d^COVID-19 blood panel comprised neutrophils, lymphocytes, neutrophil:lymphocyte ratio, C-reactive protein, Lactate Dehydrogenase, D dimer, Ferritin and high sensitivity cardiac troponin I

## Discussion

The impact of COVID-19 on healthcare systems worldwide has been vast and unprecedented. Mortality rates are high in hospitalised patients [14], yet in other patients, symptoms are mild, with some patients pauci or asymptomatic [15]. We aimed to better understand pathogenic inflammatory mechanisms that drive a more severe clinical picture by analysing cytokine levels at the point of diagnosis to help facilitate a more precision medicine approach to management in this novel disease.

Our patient group was representative of patients with SARS-CoV-2 infection admitted to an acute hospital, with a mortality rate of 18%, rising to 28% in those admitted to intensive care, presenting to hospital a week into their illness. We demonstrated that a rapid POCT for diagnosis and stratification is deliverable within the acute hospital setting, reflecting the experiences of that seen in other countries using the same cytokine platform [16].

We examined the predictive ability of demographics, standard clinical sampling and our cytokine panel. The correlation between specific cytokines and clinical inflammatory parameters was moderate at best. Indeed the moderate correlation between IL-6 and CRP was not surprising given IL-6 increases CRP transcription [17]. When the cohort as a whole was interrogated, the standard clinical demographics and NEWS2 score were accurate at predicting adverse outcome in patients with COVID-19 needing hospitalisation, with increasing age adding the greatest weight to this model. This is not unexpected and reflected in the literature, where in a large Wuhan cohort, older age, LDH and greater radiological change on computer tomography were predictive of poor outcome [18], and supports the use of the routine clinical tests that have evolved alongside our understanding of this novel disease in early stratification of patient risk as a clinical COVID-19 panel.

We demonstrated that increased levels of IL-33, IL-1β, TNF, IL-6 and IL-8 were significantly associated with adverse outcome across all age ranges, with the biggest effect size seen in IL-1β and IL-33. The larger effect size seen with IL-33 and IL-1 β may in part be explained by IL-33 s membership of the IL-1 superfamily, in addition to its role as an alarmin [19]. The combined cytokine panel added better predictive value for outcome than clinical data alone (AUROC 0.85 vs 0.71). This is reflective of the emerging literature, with recent data from a New York population demonstrating significant correlation between levels of specific inflammatory cytokines and adverse outcome [16]. To our knowledge, we are the first group to demonstrate an association between IL-33 levels and outcome in hospitalised patients with COVID-19, and this may have particular translational relevance in the context of the ACCORD2 study [20]. In addition, unpublished data from a German cohort demonstrated increased IL-33 expression in response to SARS-CoV-2 peptide stimulation in seropositive adults and an increase in IL-33 producing cells correlating with disease severity. Together these findings suggest that IL-33 has an important role in COVID-19 pathogenesis and immune response. Improved understanding of the driving factors in an individual patient’s inflammatory responses to COVID-19 offers the potential for precision medicine, such as postulated by Behrens et al. [21].

Based on the demonstration in our initial analysis that age is an unmodifiable variable with huge predictive weight in terms of adverse outcome, and to modify disease outcomes treatment targets must address modifiable areas, we then looked at patients for whom advanced age was a pre-morbid risk. In those less than 70 years, outcome is still widely variable across the population, raising the potential for identification of modifiable factors that are predictive of poor outcome and potentially amenable to novel treatment strategies. We demonstrated that TNF and IL-33 levels were both independently significant predictors of the composite outcome for the adjusted model, adding a greater degree of prediction than that of the demographic model and NEWS2 score alone. Identification of increased IL-33 and TNF levels as significantly predictive of adverse outcome is of potentially great clinical benefit, with both an anti-IL-33 and anti-TNF in treatment trials in the UK [20, 22]. The data presented here demonstrate that expression of these cytokines vary within patients with acute SARS-CoV-2 infection, and higher levels are predictive of poor outcome. Furthermore, the combined cytokine panel together increased the predictability of the model beyond that demonstrated with single cytokines.

The results suggest that cytokine analysis at the point of diagnosis has potential to improve clinical care in COVID-19, and shift management of this heterogenous disease towards precision medicine. Dexamethasone has been demonstrated to reduce mortality in those patients requiring organ support in the form of oxygen supplementation, but the mechanism remains unclear and maybe through suppression a number of different inflammatory processes in different COVID-19 endotypes. Early analysis of cytokine levels in patients with COVID-19 may help stratify those who may benefit from enrolment into treatment trials with specific targeted biological treatments that provide more focussed immunosuppression than steroids can offer, in a similar way in which immunomodulatory treatments have revolutionised asthma management beyond widespread suppression of inflammation. Furthermore, in asthma, targeted biological treatments such as anti-IL-5 medications failed to demonstrate clinical efficacy in a heterogeneous group of patients [23, 24], and it is only when targeted at those patients with elevated eosinophils as a marker of IL-5 driven inflammation that clinical benefit was shown [25]. Similar may be seen in COVID-19, which is already showing itself to be diverse in severity and presentation, and it may be that adoption of routine cytokine analysis at the point of diagnosis is key to appropriate treatment delivery moving forwards.

There are a number of limitations to our study. Our cohort was a small, single-centre study, and further work should include validation in a larger multicentre trial. However, given significance was seen despite small numbers, this suggests that the results demonstrated here could be extrapolated to a broader population. Demographic data and the COVID-19 blood panel provided a good degree of predictability for poor outcome without the addition of cytokine measures, and some may question the added benefit of cytokine analysis. This is not unexpected and reflective of the literature, as reviewed by Kermali et al. [26]. However, the cytokines and demographic data alone added a greater degree of prediction to the model than the COVID-19 blood panel and demographic data. Furthermore, the identification of individual cytokines as a predictor for adverse outcome adds additional benefit in the context of viral response endotyping and potential stratification of patents for targeted immunomodulating drugs. The generally weak correlation of clinical blood results with cytokine levels suggests that this would not be feasible from clinical blood tests alone. Although some of the cytokine levels demonstrated in this cohort fall below the lower limit of quantification for the Ella™ platform, we have internally validated these results as described. Finally, and of relevance to the broader COVID-19 literature, the lungs are a separate compartment to the blood and investigation of cytokine levels in the respiratory tract may also be beneficial in better understanding of the pathophysiology driving morbidity and mortality in COVID-19.

## Conclusions

Measurement of an inflammatory cytokine profile at the point of diagnosis is feasible in an acute hospital setting. A combined cytokine panel improves the accuracy of the predictive value for adverse outcome beyond standard clinical data alone. Identification of specific cytokines that predict poor outcome via may help stratify patients towards trials of specific immunomodulatory treatments to improve outcomes for these patients.

## Supplementary information

**Additional file 1.**

## Data Availability

All data generated or analysed during this study are included in this published article [and its supplementary information files]**.**

## Abbreviations

AUROC: Area under the receiver operator characteristic curve
CI: Confidence intervals
COVID-19: Coronavirus disease 2019
CRP: C-reactive protein
ELISA: Enzyme-linked immunosorbent assays
GM-CSF: Granulocyte-macrophage colony-stimulating factor
HS: High sensitivity
IFN: Interferon
IL: Interleukin
IQR: Interquartile rage
LDH: Lactate dehydrogenase
NEWS2: National Early Warning Score 2
OR: Odds ratio
POCT: Point-of-care test
SARS-CoV-2: Severe acute respiratory syndrome coronavirus 2
TNF: Tumour necrosis factor
