# Supplementary material for: Inflammatory phenotyping predicts clinical outcome in COVID-19
Source: Respir Res. 2020 Sep 22;21:245. doi: 10.1186/s12931-020-01511-z (PMC7506817; doi:10.1186/s12931-020-01511-z)
Supplement: Supplementary file 1 — Additional file 1. [file 12931_2020_1511_MOESM1_ESM.docx]

**Online Data Supplement**

## Inflammatory phenotyping predicts clinical outcome in COVID-19

Burke, H.*^1,2^, Freeman, A.*.^1,2^, Cellura D.C. ^1^, Stuart, B.L.^3^, Brendish, N.J. ^1,2^, Poole, S.^1,2,4^, Borca, F.^2,5^, Phan H.T.T.^3,5^, Sheard, N.^2^, Williams, S.^2^, Spalluto, C.M.^1^, Staples, K.J.^1,2,3,6^, Clark, T.W.^#1,2,3,7^,Wilkinson, T.M.A. ^#1,2^ on behalf of the REACT COVID investigators†.

*Joint First Author

^#^Joint Last author

**Corresponding author:** Dr Hannah Burke, Wellcome Trust Clinical Research Fellow.

Address: LF13A, South Academic Block, Southampton General Hospital, Southampton, SO16 6YD.

Tel: 0044(0)023816397

Email address: H.Burke@soton.ac.uk

**Table E1.** Association with serum cytokines and gender

|  | **Females** | | **Males** | | **Mann-Whitney U test (p-value)** |
| --- | --- | --- | --- | --- | --- |
|  | Mean (SD) | Median (LQ, UQ) | Mean (SD) | Median (LQ, UQ) |  |
| IL-6 | 68.88 (75.91) | 42 (22.7, 84) | 188.77 (471.09) | 50.2 (31.5, 103.0) | 0.14 |
| TNF | 24.77 (30.58) | 17.6 (12.5, 22.5) | 22.12 (11.22) | 20.3 (16.3, 25.6) | 0.17 |
| IL-8 | 111.36 (448.45) | 37.7 (21.5, 50.1) | 54.69 (52.08) | 34.7 (26.6, 63) | 0.46 |
| IL-1β | 1.04 (4.34) | 0.32 (0.20, 0.47) | 0.47 (0.32) | 0.42 (0.29, 0.62) | 0.06 |
| GM-CSF | 3.44 (9.88) | 1.25 (0.66, 2.41) | 2.43 (4.76) | 1.4 (0.85, 2.21) | 0.51 |
| IFN-γ | 17.62 (18.40) | 11.4 (3.38, 27.5) | 31.00 (65.34) | 13.4 (3.8, 27.3) | 0.77 |
| **IL-33** | 0.45 (0.49) | 0.38 (0.15, 0.50) | 0.67 (1.55) | 0.35 (0.18, 0.65) | 0.80 |
| IL-10 | 23.29 (37.79) | 14.3 (7.02, 24.2) | 27.35 (33.84) | 16.3 (10.2, 30.8) | 0.15 |

Cytokine values have been standardised before including in the model

**Table E2.** Association with serum cytokines and age

|  | Aged 70 years or over | | Age under 70 years | | Mann-Whitney U test (p-value) |
| --- | --- | --- | --- | --- | --- |
|  | Mean (SD) | Median (LQ, UQ) | Mean (SD) | Median (LQ, UQ) |  |
| IL-6 | 123.23 (333.70) | 47.25 (25.6, 89.2) | 178.48 (446.19) | 58.35 (36, 179) | 0.14 |
| TNF | 18.84 (9.17) | 18.55 (14.2, 22.5) | 31.52 (32.18) | 22.4 (17.6, 36) | **0.006** |
| IL-8 | 46.91 (45.78) | 34.6 (22.2, 55.2) | 134.81 (479.00) | 42.45 (29, 69.4) | 0.18 |
| IL-1β | 0.40 (0.30) | 0.35 (0.20, 0.54) | 1.27 (4.63) | 0.45 (0.30, 0.66) | 0.07 |
| GM-CSF | 2.23 (4.52) | 1.28 (0.87, 2.21) | 3.98 (10.58) | 1.43 (0.83, 2.34) | 0.64 |
| IFN-γ | 27.08 (54.06) | 14.45 (5.2, 29) | 23.27 (50.14) | 9.18 (2.14, 21.9) | 0.22 |
| IL-33 | 0.61 (1.47) | 0.36 (0.15, 0.60) | 0.53 (0.66) | 0.36 (0.20, 0.69) | 0.8 |
| IL-10 | 26.58 (35.73) | 15.15 (9.45, 26.5) | 24.20 (34.93) | 15.65 (8.47, 26.9) | 0.79 |

Cytokine values have been standardised before including in the model

**Table E3.** Association with serum cytokines and blood inflammatory indices (Spearman’s correlation coefficient).


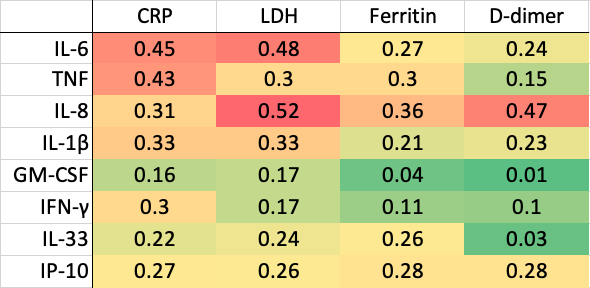


Cytokine values have been standardised before including in the model

**Table E4.** Association between serum cytokines and death

|  | Univariate Odds Ratio (95% CI) for Death | | Adjusted* Odds Ratio (95% CI) for Death | AUROC for adjusted* model | |
| --- | --- | --- | --- | --- | --- |
| Model with demographics +NEWS2 only |  |  | | | 0.93 |
| IL-6 | 1.00 (1.00, 1.00; p=0.06) | 1.00 (1.00, 1.00; p=0.099) | | | 0.94 |
| **TNF** | **1.05 (1.01, 1.08; p=0.023)** | 1.01 (0.98, 1.05; p=0.4) | | | 0.93 |
| **IL-8** | 1.01 (1.00, 1.02; p=0.056) | **1.02 (1.00, 1.03; p=0.045)** | | | 0.95 |
| **IL-1β** | **14.20 (2.08, 97.10; p=0.007)** | **79.77 (1.61, 3957.25; p=0.03)** | | | 0.95 |
| GM-CSF | 1.06 (0.99, 1.14; p=0.111) | 1.15 (0.96, 1.37; p=0.13) | | | 0.94 |
| IFN-γ | 1.00 (0.99, 1.01; p=0.507) | 1.01 (1.00, 1.02; p=0.18) | | | 0.94 |
| IL-33 | 1.14 (0.82, 1.57; p=0.45) | 1.14 (0.72, 1.81; p=0.6) | | | 0.94 |
| **IL-10** | **1.01 (1.00, 1.03; p=0.021)** | **1.02 (1.00, 1.04; p=0.039)** | | | 0.95 |

Cytokine values have been standardised before including in the model

*Adjusted models control for gender, age, NEWS2 Score, treatments (e.g. systemic glucocorticoids), comorbid conditions and smoking status.
